# Activity, socket fit, comfort and community participation in lower limb prosthesis users: a Cambodian cohort study

**DOI:** 10.1186/s12984-022-01021-7

**Published:** 2022-05-02

**Authors:** Laura Diment, RaksmeyMutta Nguon, Sovansereyrathna Seng, Vannsnavy Sit, Ply Lors, Phearsa Thor, Samedy Srors, Sisary Kheng, Malcolm Granat, Maggie Donovan-Hall, Peter Worsley, Alex Dickinson

**Affiliations:** 1 University of Southampton, Southampton, UK; 2 Department of Prosthetics and Orthotics, Phnom Penh, Cambodia; 3 University of Salford, Salford, UK; 4 Exceed Research Network, Lisburn, UK

**Keywords:** Lower-limb prosthesis, Outcome measures, Low resourced country, Accelerometer, 3D scanning, Activity, Community participation, Comfort, Limb volume

## Abstract

**Background:**

After amputation, many people become less active, feel lonely and lose independence. Understanding the factors associated with low physical activity levels and participation could contribute to defining key interventions which can support prosthesis users so they can live a more active and socially included lifestyle. This longitudinal observational study aims to assess relationships between physical activity, community participation, prosthetic fit, comfort and user satisfaction using actimetry, 3D scans and questionnaires in a Cambodian cohort of established lower limb prosthesis users.

**Methods:**

Twenty participants (5F:15M, nine transfemoral, eleven transtibial, 24–60 years old and 3–43 years since amputation) were recruited. They completed a questionnaire which included their demographics, community participation, prosthesis satisfaction and comfort at the start of the study, and between three and six months later. Their prosthetic sockets and residual limbs were 3D scanned at the start and end of the study. Accelerometers were embedded under the cosmesis on the shank of the prosthesis, to collect ten weeks of activity data.

**Results:**

Participants averaged 4470 steps/day (743–7315 steps/day), and wore their prosthesis for most waking hours, averaging 13.4 h/day (4.5–17.6 h/day). Self-reported measures of activity and hours of wear correlated with these accelerometer data (Spearman’s rho r_s_ = 0.59, and r_s_ = 0.71, respectively). Participants who were more active wore their prosthesis for more hours/day (Pearson r = 0.73) and were more satisfied with socket fit (r_s_ = 0.49). A longer residual limb correlated with better community participation (r_s_ = 0.56) and comfort (r_s_ = 0.56). Self-reported community participation did not correlate with a person’s activity level (r_s_ = 0.13), or their prosthesis comfort (r_s_ = 0.19), and there was only weak correlation between how important the activity was to an individual, and how often they participated in it (r_s_ = 0.37). A simple 0–10 scale of overall comfort did not provide enough detail to understand the types and severity of discomfort experienced.

**Conclusion:**

Associations between perceived and measured activity levels correlated with socket satisfaction in this cohort of people with established lower limb amputations. The small sample size means these correlations should be interpreted with caution, but they indicate variables worthy of further study to understand barriers to community engagement and physical activity for prosthesis users in Cambodia, and potentially in other settings.

## Background

A goal of prosthetic rehabilitation is to enable a person to perform everyday activities and engage in an independent lifestyle that meets their expectations and socioeconomic needs [1, 2]. Therefore, physical activity and community participation are key measures of an effective prosthetic service [1, 3]. However, many individuals with amputations become increasingly dependent on family and friends and can feel socially isolated [4]. It is important to understand and address barriers that may prevent prosthesis users from gaining their desired independence and social engagement, to improve the support provided by physical rehabilitation services.

The primary barriers vary between individuals, cultures, and environments. Many studies have assessed physical and service limitations, such as prosthesis functionality, the accessibility of the environment, and access to ongoing rehabilitation and support. However, there is growing recognition of the importance of the social barriers to participation. In some cases, individuals with limb absence experience social exclusion, for example not being invited to events because the venue or activity is deemed inaccessible, or because people are embarrassed by disability [5, 6]. Negative and inflexible attitudes can lead to low self-image and prevent people with amputation from becoming physically active, gaining independence and participating in community activities [7].

With a view to considering technologies to enhance access to prosthetics services, and measuring their impact, a preliminary study showed clinicians believed that incorporating digital technologies in their workflows, such as 3D scanners and accelerometers, could allow assessment of prosthesis fit and health outcomes [8]. 3D scanners are increasingly used in clinics as part of CAD/CAM socket production workflows, and scanning may also be used for creating a digital record of manually-produced sockets, and for measuring residual limb volume changes. 3D scanners are fast [9], and provide high reliability between sessions [10], but little research has used scans for assessing how gradual changes in residual limb shape influence socket fit. Accelerometers have been used in research to monitor the activity of prosthesis users in their community but only over relatively short time periods, and the technology is not in standard clinical use. [11].

This paper investigates physical, service, and social limitations to an active and socially engaged lifestyle faced by prosthesis users in and around Phnom Penh, Cambodia, through an observational cohort study. We explore how data collected from 3D scanners and activity monitors correlate with insights from questionnaires and diaries to understand prosthetic fit and comfort, as well as the individual’s community engagement, activity levels, goals, and how they feel their society perceives them. The aim is to provide insights into the factors which might contribute to enabling prosthesis users in Cambodia to live more active and socially included lifestyles.

## Methods

Ethical approval was granted by the Cambodian National Ethics Committee for Health Research (No. 311, NECHR) and the University of Southampton Ethics and Research Governance Office (ERGO45577). Study inclusion criteria were age over 18 years, use of a lower-limb prosthesis for at least two years, unilateral or bilateral amputation, having a prosthesis in good working order, able to walk for at least 1hr with rests as necessary, having no other complex or life-threatening comorbidities, and being able to give informed consent to participate. Approval was granted to recruit a convenience sample of 20 participants, selected purposively for a balance of transfemoral and transtibial amputation levels, who were invited to consider participation during their routine consultation. Four Cambodian Prosthetic and Orthotic student researchers (RMN, SRS, VSS, PL) provided verbal participant information in Khmer. Due to varying participant literacy, the researchers used a video narrated in Khmer to explain the protocol, demonstrate how the accelerometers and 3D scanners work and explain data protection. Participants made two visits for data collection, following guidelines published by the International Society for Prosthetics (ISPO) and the Exceed Research Network (ERN) for ethical conduct of mobility assistive technology research in low resourced settings [12]. The study was designed for data collection at two time points (baseline and after three months) for all of the following measures except activity monitoring, for which a single ten-week dataset was collected between the two visits.

### Data collection

Each participant completed a questionnaire on demographics, community participation, activity, society perceptions, and prosthesis satisfaction, comfort and fit. The questionnaire included aspects of the TAPES-r tool, which analyses a prosthesis user’s psychosocial adjustment, activity restriction, and prosthesis satisfaction [13]. However, the TAPES-r did not cover all issues of interest in this study around occupation, community participation, activity importance, types of discomfort, and specifics on the prosthesis design, socks and liners. Furthermore, not all TAPES-r questions were relevant to the research question or to prosthesis users in Phnom Penh. Therefore, a subset of TAPES-r questions was included, and additional questions were constructed (Appendix 1) in the same format of short-form answers and Likert-scales of between three and eleven points.

The participants’ reported community participation activities were categorised based on the World Health Organization’s (WHO) International Classification of Functioning, Disability and Health (ICF) [14], and categories coded by Hordacre et al. [1, 15] and by Chang et al. [16]. Thus, activity was condensed into seven categories (Table 1) that differentiate between the *levels of physical exertion* required, and whether the activities are considered *responsibilities* (e.g. providing for oneself and ones’ family) or *liberties* (e.g. leisure and entertainment). Based on the TAPES-r and the Community Participation Indicators (CPI), participants were asked whether they are able to participate in each category of community participation, how often, how important the activity is to them and whether they use their prosthesis for the activity. Participants were also asked about key lifestyle activities to give an indication of their physical capabilities, to list any other reasons they regularly leave the house, and any activities that they would like, but are physically unable, to complete. To indicate barriers to community participation, participants were also asked how strongly they agree or disagree with statements such as *‘my community treats me with respect and as an equal’*, based on TAPES-r and CPI questions. To augment the TAPES-r questions on prosthesis satisfaction, participants selected how much they had experienced different types of residual limb discomfort [17] in the previous month (aching muscles, skin tenderness, sudden pain, rubbing, itchiness, heat and sweat, numbness and phantom limb pain) and listed any medications they take for pain, and the frequency of use.

**Table 1:** Community participation categories, and their indicative physical exertion level and description as responsibility or liberty

Category	Physical exertion (low/high)	Considered a responsibility or a liberty
Paid work, employment and education	Low or high	Personal and family responsibility
Relationships and social interactions	Low	Liberty but sometimes a social responsibility
Economic life (shopping, banking etc.)	Low but can include carrying	Personal and family responsibility
Physical activity, exercise and sport	High	Liberty but sometimes a personal responsibility
Religion and spirituality	Low	Liberty or personal responsibility
Healthcare	Low	Personal responsibility
Leisure and entertainment	Typically low	Liberty

**Fig. 1:**
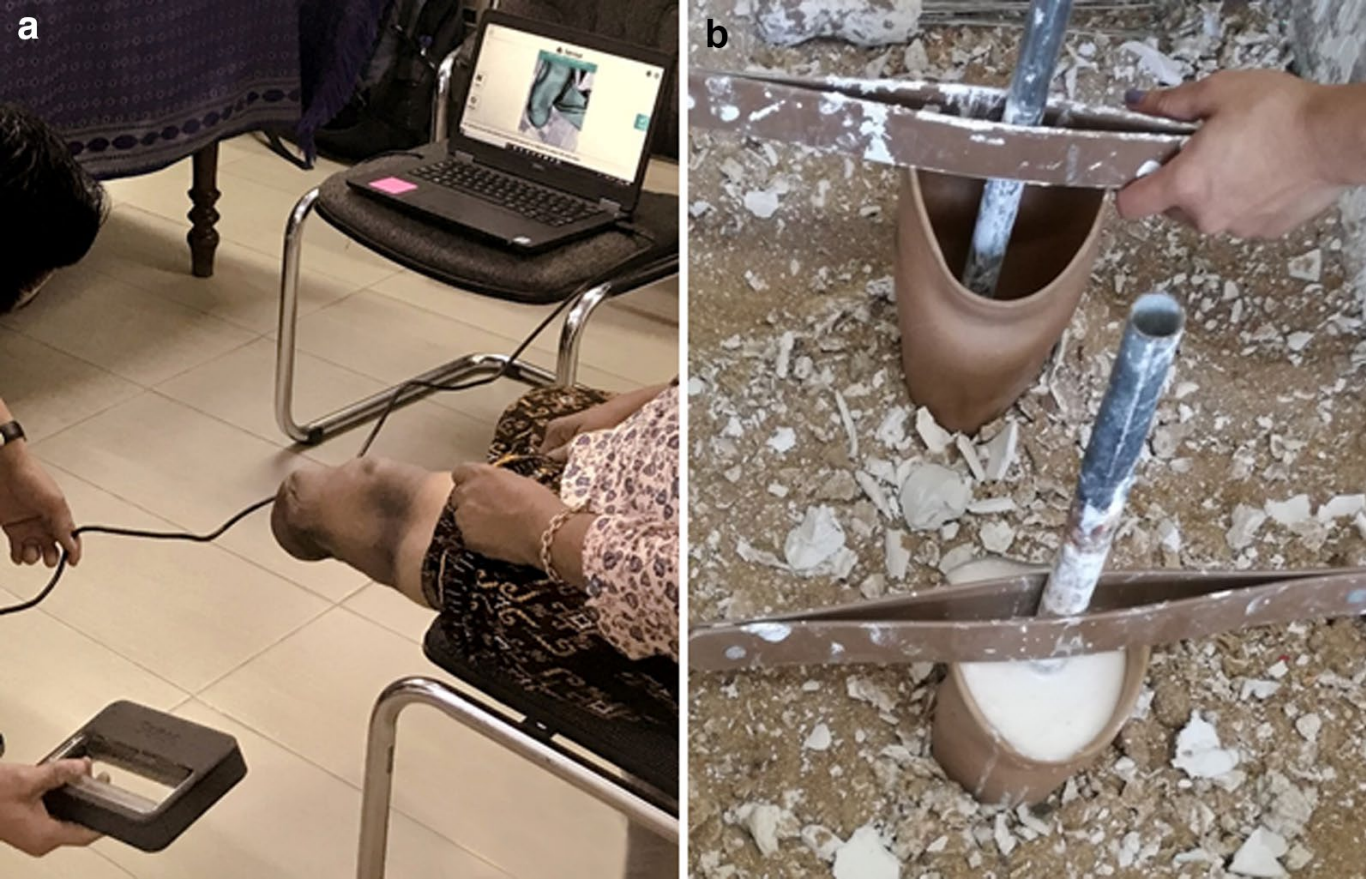
**A** 3D scanning a participant’s residual limb, and **B** creating alginate moulds of the sockets

Shortly after doffing the prosthesis, participants had their residual limb 3D scanned using a Sense scanner (3D Systems, South Carolina) (Fig. 1A), which has shown 95% repeatability to less than 79 ml for residuum volumes and 15 mm for perimeters [18]. An alginate mould was created from their socket (Fig. 1B) and scanned to enable measurement of volumetric socket fit using ampscan open-source software [19], which provides a map of the shape differences between two scans, as well as information on their cross-sectional area, perimeter, sagittal width, coronal width and volume.

**Fig. 2:**
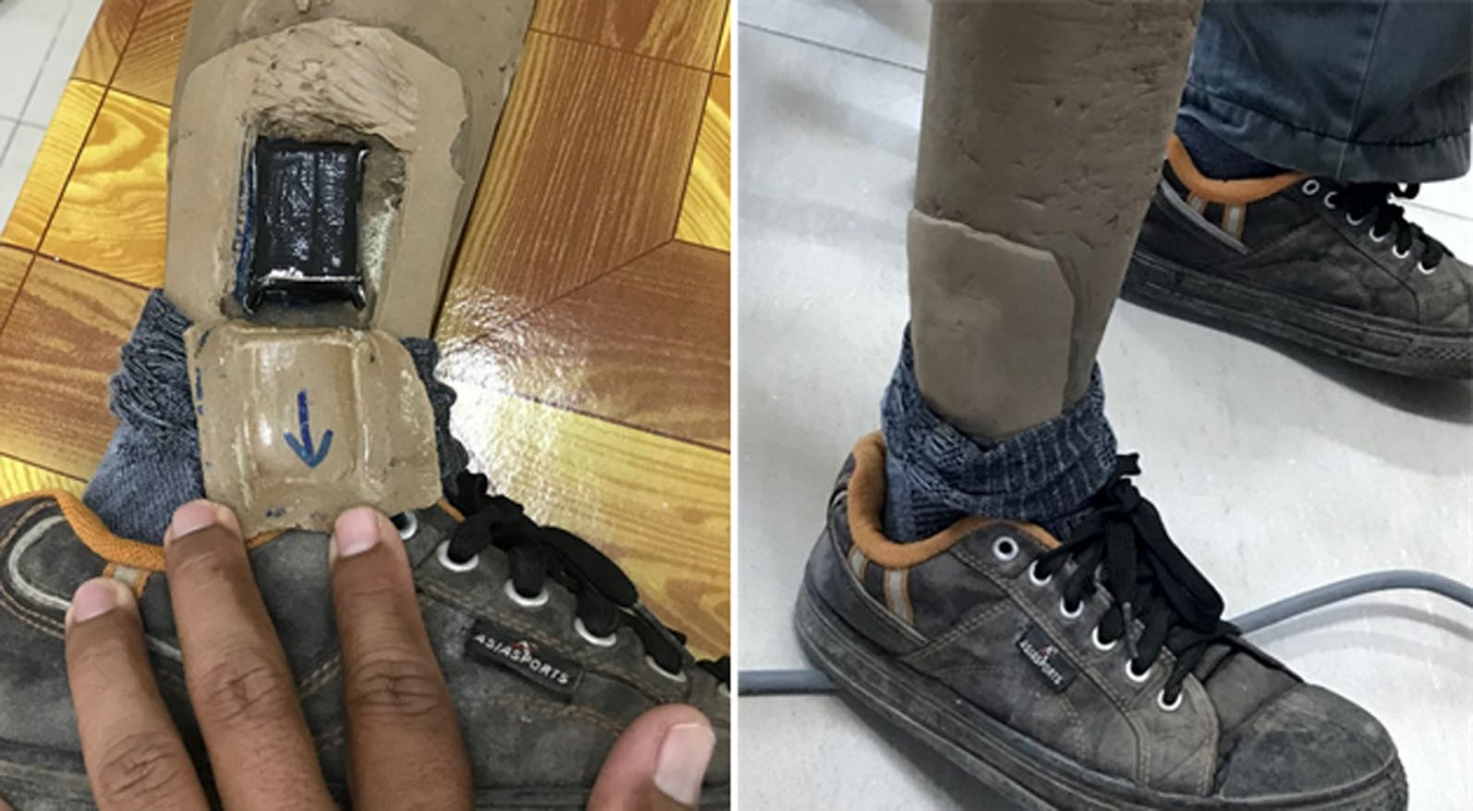
Embedding the accelerometer in the prosthetic cosmesis. A new cosmesis was made and provided following study completion

An AX6 accelerometer (Axivity, Newcastle, UK) was used to collect triaxial accelerations at 25 Hz for ten weeks, which were converted to estimated activity parameters. The sensor was embedded into the prosthesis on the lateral side of the shank (Fig. 2), using a standardised orientation. Embedding the sensor addressed concerns raised during preliminary consultation by making it unobtrusive, as well as adding to its protection and reducing noise in the signal associated with movement artefacts which might result from mounting on the skin. Participants consented to have their cosmesis adapted to accommodate the accelerometer, with the knowledge that the cosmetic layer would be replaced after the study.

Participants completed an hourly timetable of their average week at the start and end of the study, and took home a diary to register times when they left the house and the reason for leaving (Appendix 2). The timetable and diary were compared to the data collected from the accelerometer to assess correlation between measured and self-reported activity.

During a second visit, the accelerometers were removed and a new cosmesis provided, the residual limbs were re-scanned, and participants filled in the questionnaire and timetable again. Questions on how the coronavirus affected their lifestyle and how they felt about the 3D scanner and accelerometer were also included (Appendix 1).

#### Data analysis 1: Patient-reported outcomes

##### Questionnaire and timetable data

Scores were created from the questionnaire and timetable for separate topics of (i) self-reported hours of wear, (ii) self-reported activity, (iii) community participation, (iv) social perceptions, (v) prosthesis satisfaction, (vi) comfort, (vii) fit, and (viii) number of socks and liners worn, by collating and summing the scores for each relevant question within a topic. Ordinal data was ranked from lowest to highest in each category.

*Self-reported hours of wear* were calculated using the average number of hours/day the prosthesis was worn from the participants’ two average-week timetables.

*Self-reported activity* was calculated by assigning each activity in the weekly timetable a metabolic equivalent (MET) score based on the Adult Compendium of Physical Activities (Appendix 2, Table 3) [20]. MET scores for the start and end of the study period were averaged.

*The community participation score* was created based on the frequency of activity for the seven categories (Table 1). All questionnaire-based scores were calculated from the questions weighted equally except the *societal perceptions score*, where ‘*I don’t mind people asking about my prosthesis’* was used as a differentiator on the main question of interest ‘*My community treats me with respect and as an equal’,* to provide unique participant ranking.

The *prosthesis satisfaction score* was a summation of the scores for each category of appearance, weight, usefulness, durability, fit and comfort of their prosthesis. In addition, specific elements of this questionnaire were used to identify comfort and socket fit. These sub-sections were selected as they represent primary considerations for clinicians and prosthesis users, and they allowed assessment of how well a single score correlates with a more detailed scan-based analysis. A *combined discomfort score* was created using responses to the types and severity of discomforts experienced in the past month, with participants reporting lower discomfort assigned a higher ranking. The average *number of socks and liners* worn was used as an additional indicator of socket fit, with three socks weighted equal to one liner, based on standard sock and liner thicknesses.

##### Data analysis 2: Technology-derived measures of socket fit and activity

**Fig. 3:**
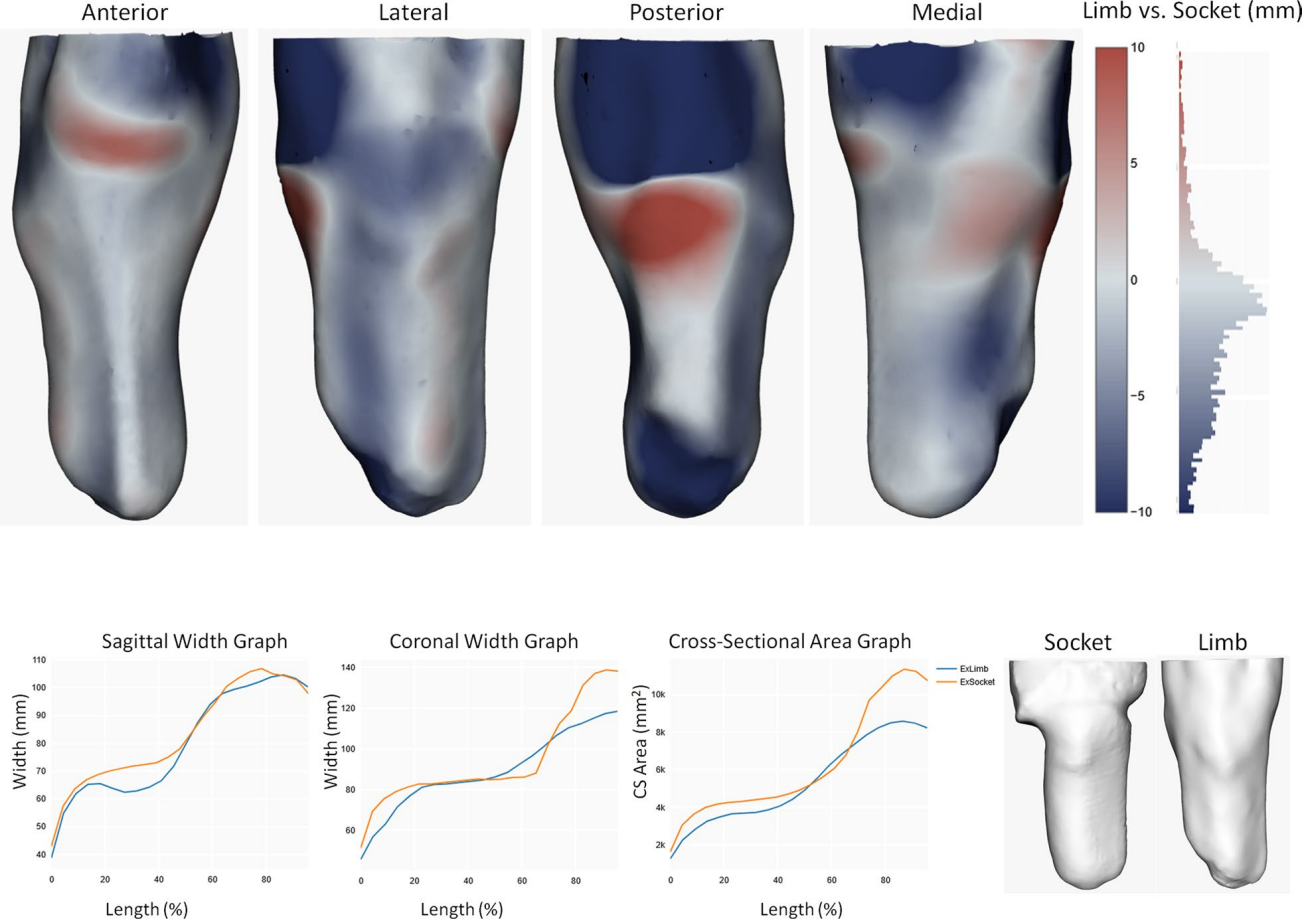
Ampscan compares two 3D scans. This example compares a participant’s socket to the residual limb shape at the start of the study. This shows local press-fit rectifications at the patellar tendon (anterior), the posterior calf/popliteal fossa (posterior), the medial tibial flare, and the tibialis anterior muscle (lateral). The socket is spaced away from the limb at the fibula head (lateral) and the distal posterior surface where the limb has a concave scar geometry. Comparisons between the two shapes are made for volumes, and profiles of cross-sectional area, sagittal width and coronal width vs. % length

Using a previously reported procedure [18], the 3D scan of the limb at the start of the study was aligned with the socket scan by automatic iterative-closest-point (ICP) surface matching and manual adjustments by an experienced observer (LED) (Fig. 3). The limb scan from the study end was then aligned with the limb scan from the study start.

*Limb volume* was then estimated from the 3D scan taken of the limb at the start of the study, and the magnitude of *limb volume fluctuation* was reported as the percentage difference between the volumes calculated from scans at the beginning and end of the study (at 0 and 3 or 6 months). The *closeness of socket fit* was estimated using the distance between each mesh vertex on the 3D scan of the limb from the start of the study, and its corresponding mesh node on the socket scan. The median distance was used so that local socket rectifications were excluded from consideration. The *level of amputation score* ranked participants from shortest to longest residual limb length, as higher amputation levels typically correlate with poorer outcomes [21]. A transfemoral residuum was ranked 1–3 from short to long, a knee disarticulation was ranked 4, and transtibial residua were ranked 5–7.

For *objective activity* measures, the average *steps per day* were calculated from the accelerometer data over the ten-week period of acquisition. The *hours of wear* were easily observed from the accelerometer data, with step count and activity dropping to zero immediately when the prosthesis was removed. The daily *hours of non-wear* were averaged across the ten weeks. The accelerometer data were compared to the *self-reported* timetables.

#### Statistical analysis

The data were assessed for normality prior to descriptive statistics and analysis. All series variables were found to be normally distributed using the Kolmogorov–Smirnov test, so a Pearson Correlation (r) was used to assess correlations. Correlations with ordinal variables were determined using Spearman Rank Correlation (r_s_) along with comparisons between the demographic data on the level of amputation with age, years since amputation and time since the socket was last fitted, and data from the 3D scans and accelerometers.

## Results

### Demographics

In January 2020, 20 participants volunteered to participate in the study, but only ten had started when it was halted by the COVID-19 pandemic. These ten participants were followed up at six months. A further ten joined the study in October 2020 and were followed up at the originally-planned three months. The participants all had unilateral amputations, which were nearly evenly split between transtibial and transfemoral levels, and the predominant reason for amputation was trauma (Table 2). Participants covered a wide range of ages and time since amputation, and a range of professions, ranging from occasional (about once per month) to daily work.

**Table 2:** Participant demographics

Descriptor	Demographics	
Level of amputation	Transfemoral	9
	Transtibial	11
Sex	Female	5
	Male	15
Age (years)	Mean 50 (range 24–60)	
Time since amputation (years)	Mean 26 (range 3–43)	
Reason for amputation	Trauma (landmine)	11
	Trauma (traffic accident)	7
	Cancer	1
	Infection	1
Profession	Taxi driver	5
	Farmer	4
	Vendor	4
	Engineer	1
	Community worker	1
	Police officer	1
	Beautician	1
	Retired veteran	1
	Security guard	1
	Unemployed	1
Work frequency	Daily	7
	Once per week	2
	Occasionally	11

### Correlations

**Fig. 4:**
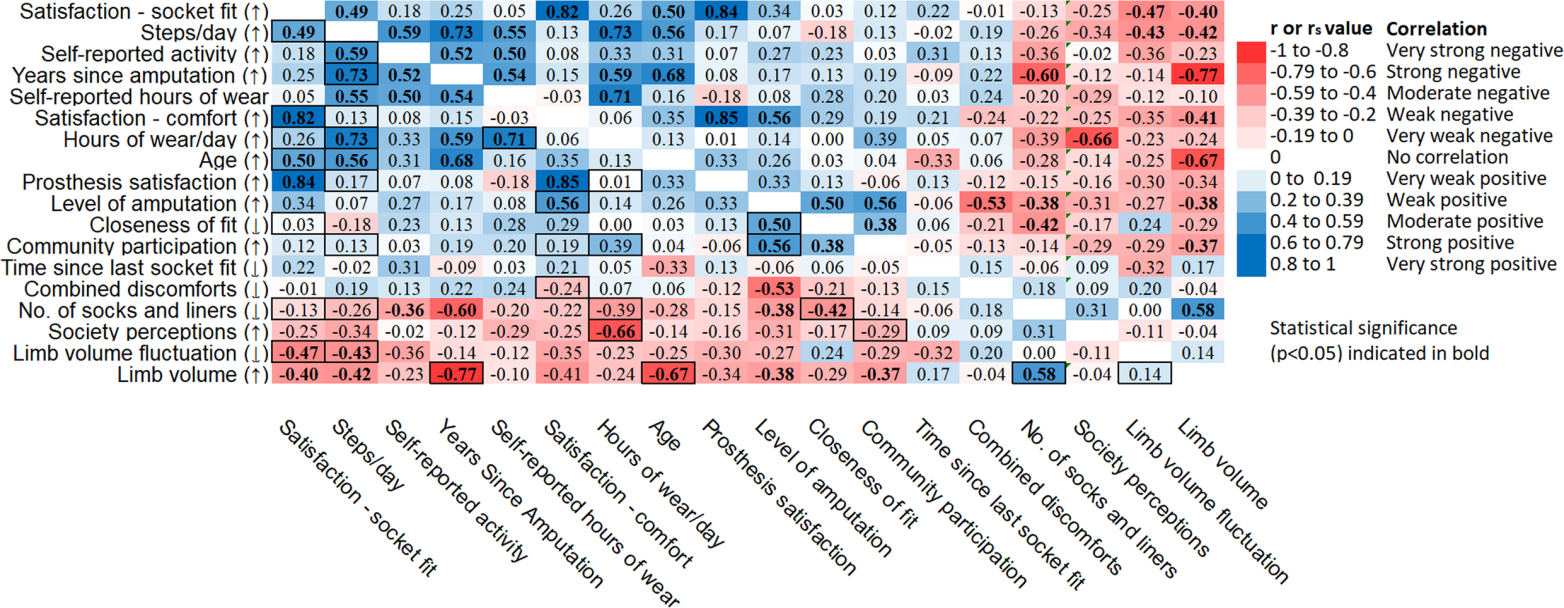
Correlation matrix of key variables. Pearson coefficients (r) are given for correlations between series variables, and Spearman rho (r_s_) are given for correlations with at least one ordinal variable. The bordered cells are correlations of particular interest, which are discussed in greater detail in the text. Statistically significant correlations (p<0.05) are indicated in bold. The up and down arrows show the direction at the positive end of the scale. For example, more satisfaction for comfort was ranked higher (↑), and more discomfort was ranked lower (↓). Variables are ordered by their absolute correlation score summed across all comparisons

A two-tailed paired t-test between all scored questionnaire responses at the start of the study and responses at the end of the study showed no significant difference in responses over time (p = 0.248), so for the correlation scores, the average of the two measures was used. The scores were plotted in a Correlation Matrix (Fig. 4) providing a visual overview of the associations between outcome measures, colour coded depending on the strength and nature of the association.

### Activity levels

**Fig. 5:**
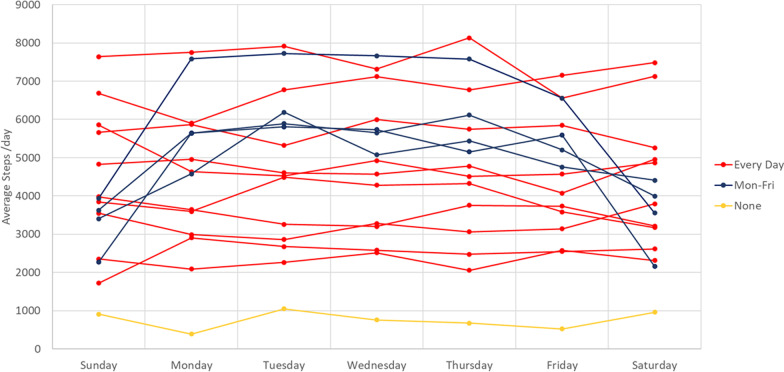
Average steps across the ten weeks for each participant, divided into days of the week, categorised by working patterns

Seventy days of accelerometer data were collected successfully for 15/20 participants. Participants wore a device on average for 13.4 h/day (range 4.5 to 17.6 h/day). Most wore their prosthesis every day from morning to night, which was consistent with their questionnaire and timetable reports. A strong positive correlation (Pearson r = 0.71) was observed between self-reported hours of wear and hours of wear recorded by accelerometer (Fig. 4). People who walked more steps/day wore their prosthesis for longer each day (r = 0.73). There was moderate positive correlation between the self-reported activity (timetable and estimated MET scores) and objective accelerometery scores (r = 0.59). On average, participants took 4492 steps/day (743–7502 steps/day, Fig. 5).

### Community participation

**Fig. 6:**
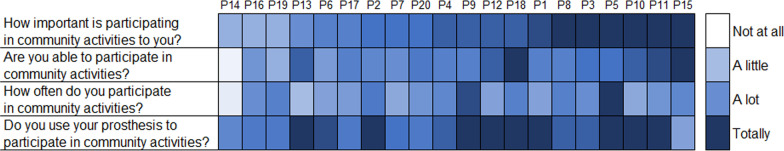
Community participation overview. Participants are ordered by their prioritisation of ‘How important is participating in community activities to you?’, showing that this had little correspondence with whether they were able, or how often, they reported participating

Community participation did not correlate with the number of steps/day (r_s_ = 0.13), but it weakly correlated with hours of wear/day (r_s_ = 0.39) (Fig. 4). Participants most regularly left the house for work and exercise, and least often for leisure or medical appointments. All participants used their prosthesis for all activities outside the home except for one, who reported exercising daily without a prosthesis. Each activity was rated by most participants as important. The category rated as least important was leisure and entertainment, and working was the most important. Notable leisure and entertainment activities listed as important but difficult or unachievable were participating in sport or exercise (10/20 experienced difficulty and four were unable to participate), visiting friends and family, and religious activities. Of the four participants who stated they were unable to leave the house for leisure and entertainment, only one felt this was important. A person’s reported importance of engaging in their community only weakly correlated with how frequently they participated (r_s_ = 0.37, Fig. 6).

Outside the seven categories of activity, participants also reported leaving the house for *“guarding someone else’s house”, “volunteering in their community”, “going to a wedding”,* and *“taking a family member to hospital”*. Participants most frequently reported wishing, but being unable to carry heavy loads. Participants also wished to climb trees, run fast, jump, fish with a net, walk long distances across uneven terrain and through water and mud, exercise, balance better, cross their legs, and work in their prior profession. Responses to how they are treated by their community were highly varied. *“My community treats me with respect and as an equal”* had both strong agreement and strong disagreement across the participants, as did *“I don’t mind people asking about my prosthesis”*.

### Comfort, satisfaction and fit

**Fig. 7:**
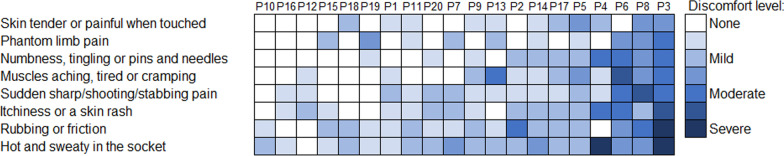
Specific discomfort scores, with participants ordered by increasing total discomfort score

All participants were relatively satisfied with all aspects of their prosthesis. All scores except one, for socket fit, comfort, weight, durability, and appearance were from 5 to 10/10. All participants rated usefulness from 8 to 10/10. The average comfort rating was 8.3/10, but participants had a range of discomforts, with 12 reporting moderate to severe discomfort in at least one category (Fig. 7). The main discomfort category experienced was heat and sweat. The sum of discomforts experienced in the previous month did not correlate with their overall satisfaction with prosthesis comfort (r_s_ = − 0.24). More years since amputation strongly negatively correlated with limb volume (r = − 0.77). People with a larger limb volume wore more socks or liners (r = 0.58), but there was no correlation between limb size and limb volume fluctuation. The number of socks and liners worn did not correlate with satisfaction with socket fit, but moderately negatively correlated with closeness of fit (r = − 0.42).

### Comparing participants with transtibial and transfemoral amputations

People with transtibial amputations wore their prostheses for longer each day than the transfemoral group (mean 13.7 vs 13.0hrs) and walked more steps per day (mean 4622 vs 4193 steps). A two-tailed paired t-test revealed no significant difference in questionnaire responses between the transtibial and transfemoral prosthesis participants (p = 0.36), so the results were analysed in a single group. However, although the overall comparison showed similar trends in both groups, there were some notable exceptions:

- Transfemoral prosthesis users who experienced less discomfort were more engaged with their community (r_s_ = 0.63), walked more steps per day (r_s_ = 0.50) and wore fewer socks and liners (r_s_ = 0.56). Transtibial prosthesis users showed no correlations between discomfort and these measures.
- Unlike participants with transtibial amputation, satisfaction with socket fit of those with transfemoral amputation also correlated with steps/day (r_s_ = 0.84), hours of wear/ day (r_s_ = 0.50) age (r_s_ = 0.60) and years since amputation (rs = 0.63), but not time since last socket fit (where transtibial prosthesis users showed a moderate correlation (r_s_ = 0.59)).
- Closeness of socket fit was very strongly negatively correlated with prosthesis satisfaction for the transfemoral prosthesis users (r_s_ = 0.82), while it was moderately positively correlated for transtibial prosthesis users (r_s_ = 0.44).

## Discussion

This observational study involving people with lower limb transfemoral and transtibial amputations in Cambodia combined patient-reported prosthetic use, activity, and social participation with technologies to objectively characterise socket fit, limb shape and step count. Correlation analysis was used to identify trends between these outcomes, which varied considerably across the participants. The heterogeneous cohort was representative of the Cambodian prosthesis user population, but its small size means the reported correlations should be interpreted with caution. However, some novel insights were gained from this first-of-kind mixed methods approach.

Most participants were highly active, generally satisfied with their prosthesis and wore it for most waking hours, and rated it as highly useful despite having various discomforts and restrictions to specific activity participation. All participants had relatively fixed daily and weekly routines according to self-reported timetables and corresponding accelerometer data. Trends between daily step counts were observed, with some participants taking fewer steps at weekends compared to weekdays, and this corresponded with their working patterns (Fig. 5). There was high consistency within an individual’s activity, but high variability between participants. Age and years since amputation were strongly correlated (r = 0.68), and the years since amputation strongly correlated with steps per day (r = 0.73). Participants reported finding their prosthesis uncomfortable, heavy and unattractive, which may account for their lack of community engagement. Those who were more active wore their prosthesis for longer periods and were more satisfied with socket fit, were longer since their amputation and showed greater variability in residual limb volume. Community participation only weakly correlated with hours of prosthesis wear and did not correlate with their activity level, or how comfortable their prosthesis was.

It is not surprising that participants who were satisfied with their prosthesis and found it comfortable were more likely to be active, but it was interesting to note that being more active did not correspond with greater community engagement. This may simply reflect individual personalities, perhaps showing that people who crave social engagement will get involved in their community if given a chance, regardless of their physical fitness and prosthetic function. When assessing community participation and enfranchisement, it is important to assess the meaningfulness of activities to the individual, as well as the amount they engage with their community. For example, in Cambodia, participation in cultural and traditional ceremonies carries very high importance [22], such as being invited to a wedding. This includes whether the individual has choice or control over the activities they participate in, and whether they are liberties or personal or societal responsibilities [6]. Anecdotally, people often consider liberties to be lower priority than responsibilities, but more meaningful.

Some individuals rated community participation as important but reported difficulty participating, which might suggest that improving the prosthesis, rehabilitation, services, and accessibility of the environment may improve their community engagement. However, most participants who rated community participation as highly important but did not participate much in their community, said that there were no barriers preventing their participation. This may indicate that they have not matched their lifestyle to the tasks they theoretically find most important, due to no fault of the prosthesis or society, or it may indicate that some of the barriers are more hidden, such as mental health, responsibilities, community expectations, and attitudes limiting their engagement. Elsewhere in the cohort, the five participants who were most engaged in their community all struggled to do a number of activities, particularly sport and exercise. Unexpectedly, societal perceptions correlated negatively with hours of wear, steps per day and community participation (r_s_ = − 0.66, − 0.34 and − 0.29, respectively), suggesting that people who were more active and engaged with their community were less satisfied with society’s perception.

When comparing the accelerometer results with the timetables, most physical activity related to employment and responsibilities such as shopping and child-minding, rather than liberties such as leisure and entertainment, visiting friends and family, and sport. This is consistent with Cambodian cultural norms where family is often prioritised over personal concerns [23]. This may also represent viewing leisure and entertainment from a Western lens which may be appropriate for young people in the capital, whereas our participants, from both urban and rural backgrounds, identified example leisure activities as playing chess, sightseeing, singing karaoke or watching TV [24]. Older age and time since amputation correlated with greater activity, which was unexpected given that all participants had at least three years since their amputation and all were established prosthesis users. This may indicate that it takes longer in this population for the residual limb tissues to mature and stabilise in volume sufficiently for confident prosthesis use than reported in previous studies [9], or that as people become more familiar with the prosthesis and normalise wearing it, their activity is promoted. Social and cultural factors could also contribute, with older Cambodians typically living more active lifestyles and working more active jobs than the younger generation. Eighteen of the 20 had regular employment, consistent with survey data from 2014 when over 70% of women and 88% of men with disabilities were employed, in the 15–49 year age band, though difficulty walking was reported as more prevalent in those who did not work. [24].

The population of people with limb-loss in Cambodia is relatively young in comparison to most high-income countries, with people experiencing their amputations at a younger age [25], which was reflected in this cohort, with no participants aged over 60. Almost all had amputations due to trauma, and were highly active, walking an average of 4492 steps per day which is in line with recommendations for moderate-to-vigorous physical activity (MVPA) for people without physical disabilities [26]. This difference in demographics of people with limb loss between countries supports the need for location- and population-specific prosthetic design and rehabilitation interventions. There was high variability in average steps per day between individuals, but more useful social insights might be identified in differences in step patterns between weekdays and weekends. Changes in these activity patterns could indicate adverse events, such as if someone has become unable to work. Activity correlated with greater variability in residual limb volume between time points of investigation, as well as in comfort. It may be that activity itself is related to residuum volume, as sweat, hydration, and musclular blood flow affect daily fluctuations and long-term changes in volume [27]. Short term, gross residuum volume reduction is seen due to recovery of oedema followed by more gradual muscle atrophy [28–30] alongside changes in tissue composition under mechanical loading with a prosthesis [31]. These studies report volume loss in the order of 10–35% in the first 18 months after amputation. However, given that this population had an average of 26 years since amputation, this change is unlikely to be noticeable when comparing scans 3–6 months apart. Furthermore, since the observed volume changes were smaller (< 10% for 17/20 participants) and did not show a systematic increasing or decreasing trend across participants, they are likely to be predominantly linked to short-term fluctuations.

As reported in previous studies [21], a longer residual limb correlated with better quality of life outcomes, such as more community participation (r_s_ = 0.56) and greater comfort (r_s_ = 0.56). Longer residual limbs also correlated with a closer fitting prosthetic socket (r_s_ = 0.50). Closeness of fit was negatively correlated with prosthesis satisfaction for transfemoral prosthesis users, but positively correlated for transtibial prosthesis users. This result may relate to the different load transfer mechanisms behind transtibial and transfemoral sockets, where transtibial sockets often interact with bony prominences and local load-tolerant soft tissues such as the patellar tendon and gastrocnemius muscles, whereas transfemoral sockets may use gross soft tissue compression over the majority of the residuum [32]. The lack of relationship between socket fit satisfaction and the number of socks and liners worn suggests the participants were successfully managing residuum volume fluctuations. Interestingly, greater limb volume fluctuation tended to correlate with better outcomes across the other measures, particularly steps per day (r = − 0.43).

All participants rated usefulness of the prosthesis very highly, despite listing activities in which they could not participate. Participants reported general satisfaction with all aspects of their prosthesis, despite 12/20 individuals reporting moderate to severe discomfort in at least one category. Overall prosthesis satisfaction and satisfaction with comfort were not found to correlate with hours of wear, steps per day or community participation. However, there was moderate positive correlation between satisfaction with socket fit and the number of steps/day (r_s_ = 0.49). Socket comfort was also very strongly correlated with satisfaction with socket fit (r_s_ = 0.82), but interestingly the overall socket comfort did not correlate with the combined individual discomforts experienced over the previous month (r_s_ = − 0.24). The fact that a general comfort score did not correlate negatively with discomfort, and that the types of discomfort were so varied, may indicate a case for more detailed questions to establish a true picture of prosthesis comfort than a simple Visual Analogue Scale (Socket Comfort Score). This observation may be linked to asking participants about overall comfort before asking questions about individual discomforts. It is possible that overall comfort would be scored differently if asked *after* listing potential discomforts, but the protocol used this order because the Socket Comfort Score is normally administered alone in clinical use.

Self-reported measures of activity and hours of wear, based on a timetable of the participant’s usual weekly schedule and estimated metabolic rates [20], provided a good estimation of the person’s activity level monitored with accelerometers. This provides some assurance that recount reliability can be used when monitoring activity with wearable devices is infeasible. However, a single question on the average hours-of-wear per day was not reliable, with most participants unable to give an approximate number, particularly if some days of the week tended to be more active than others.

### Limitations

These correlations should be interpreted with caution due to the small heterogenous sample. The analysis has highlighted variables worthy of further study to understand barriers to community engagement and physical activity for prosthesis users in Cambodia, and potentially in other settings. The participants received the prosthesis and services from Exceed Worldwide at no cost, and the study was run at the National Institute for Social Affairs’ Department of Prosthetics and Orthotics (DPO) which shares a site and staff, so there may be a bias towards a positive response to satisfaction. Some of the actimetry sensors showed errors (Participants 12, 14 and 18) or did not provide any data due to getting waterlogged (P2), and the data from one sensor was accidentally saved over another (P11). These issues were partially due to the coronavirus pandemic causing longer gaps than anticipated between sending out the accelerometers and collecting them, and the researchers being unable to supervise students in-person, to double-check all sensors were working and data stored correctly. However, after those missing actimetry datasets the group still had five female and ten male participants, seven transfemoral and eight transtibial, and the losses did not substantively change the group demographics. Similarly, there will inevitably be uncertainty associated with measures from 3D scans of residual limbs arising from variability in raw scan measurements, subjectivity in alignment, and within-day tissue shape and volume fluctuations. To minimise inconvenience on participants, repeated scans were not collected, but the observed volume changes were larger in 18/20 participants than the previously reported reliability of scans from the Sense device using the same procedure (± 1%) [18]. For the same reasons, socket pressure was not conducted in this study but this would provide another objective measure of the limb-socket interface mechanics.

Cambodia did not have many recorded cases of COVID-19 in 2020 and data were not collected during lockdowns. Almost all participants said their lifestyles and activities were unaffected by coronavirus. However, by the end of the study, five decreased their frequency of work or stopped working, and ten who had previously worked once a week or less, worked daily. One stated that they avoided leaving the house unnecessarily during the pandemic, eleven said they were less active than in the previous year, and eight said they left the house less than in the previous year. Five participants also reported that the coronavirus situation has severely impacted their finances. These results suggest that the activity and community participation data collected is relatively reliable for these participants in a normal year but should still be interpreted with caution.

Care was taken to ensure cultural appropriateness of the study, although this required a small departure from the established TAPES-r research tool by adding supplementary questions which have not been tested for validity and reliability. For the present study it was judged to be more important to get an overview of the range of factors and how they correlate with activity and community participation, than to adapt and validate the standard questionnaire for use in Cambodia, and there was a good range of variability across the responses for most questions, which means the tool successfully differentiated between individuals. Running a preliminary participant involvement study ensured the questions and methods were useful for clinicians and prosthesis users [8], and that their concerns were addressed in advance, considering for example obtrusiveness and discomfort of accelerometers, and privacy of study data.

Further research is required to test whether the trends found in this paper are true across a larger population of prosthesis users, and how we might act upon them to improve their activity and social engagement. The data revealed promising trends for the researchers and clinicians to know which scores to study in more detail and which questions might be suitable to understand an individual’s key hopes, and the issues they face. They also show the value of the accelerometers and 3D scan data for collecting useful measures, to use alongside questionnaires and physical assessments. Changes in lifestyle and hours of wear, as shown by an accelerometer, may also help with prediction of when the prosthesis is no longer supporting the person as it should. This may be obvious in settings with easy, frequent client–clinician contact, but less clear in cases where someone lives in a remote, rural community, or where there may be socioeconomic, geographical or cultural barriers to service access. The study provides valuable insights into how future prosthetic rehabilitation or device developments might be assessed against the participants’ wished-for activities, including more vigorous and challenging conditions such as carrying heavy loads and walking long distances across uneven terrains. These may be linked to enabling people to work in their profession from prior to their limb loss.

## Conclusion

This paper investigated satisfaction, social engagement and physical activity participation in lower limb prosthesis users in Cambodia, comparing self-reported and objective measures. Across a small but diverse and representative sample, participants found their prostheses highly useful, and were generally satisfied with all aspects of the prosthesis. However, overall comfort scores typically used in clinics did not reflect the range and severity of discomforts experienced, so a more in-depth way of assessing comfort may be beneficial. Likewise, a single score of hours-of-wear or Likert scales of activity were substantially less accurate than estimating activity using a timetable and metabolic rates, which was comparable to accelerometer data. Participants who were more active wore their prosthesis for longer and were more satisfied with socket fit. However, community participation only weakly correlated with hours of prosthesis wear and did not correlate with a person’s activity level or their prosthesis comfort. Indeed, people who were more active and engaged with their community were less satisfied with their society’s perceptions. More widely, the findings support continued work into the acceptance of people with disabilities across society, demonstrating the ongoing value of funding outreach programmes like community workers who use prosthetic limbs.

## Data Availability

All study data have been made openly available from the University of Southampton repository at https://doi.org/10.5258/SOTON/D2050.

## Appendix 1: Questionnaires

Additional questions at the end of the study:

Activity diary completed during the 10-week accelerometer data collection:

## Appendix 2: Energy Intensity of Activities

See Table 3.

**Table 3:** Energy intensity of activities undertaken by participants in the study (MET score) [20]

Activities	MET score
Sleeping	1
Relaxing	1
Using the TV/computer/phone	1
Religious activities	1.3
Reading	1.3
Eating	1.5
Occupations that are primarily sitting (desk job)	1.5
Occupation: beautician/hairstylist	1.8
Socialising (standing/talking/on the phone etc.)	1.8
Getting ready for the day (showering, toilet, dressing etc.)	2
Driving	2
Shopping	2.3
Occupations that are 50% sitting, 50% standing (retail, security, community development)	2.3
Occupation: police	2.5
Caring for children	2.5
Slow walking	2.5
Occupation: hygiene engineer	2.8
Housework (laundry, sweeping, mopping, sewing, etc.)	2.8
Cooking	3.3
Fishing	3.5
Occupation: Custodial work (cleaner)	3.8
Occupation: Farmer	4
Exercise	5.5
Bicycling (commuting)	6.8

## Abbreviations

CAD/CAM: Computer Aided Design and Manufacturing
CPI: Community Participation Indicators
DPO: Department of Prosthetics and Orthotics (National Institute of Social Affairs, Cambodia)
ICF: International Classification of Function
ICP: Iterative Closest Point
ISPO: International Society for Prosthetics and Orthotics
MET: Metabolic equivalent
MVPA: Moderate to Vigorous Physical Activity
PPI: Patient and Public Involvement
TAPES-r: Trinity Amputation and Prosthesis Experience Scales – Revised
WHO: World Health Organisation
